# Impact of the COVID-19 Pandemic on Changes in Consumer Purchasing Behavior in the Food Market with a Focus on Meat and Meat Products—A Comprehensive Literature Review

**DOI:** 10.3390/foods13060933

**Published:** 2024-03-19

**Authors:** Jagoda Żurek, Mariusz Rudy

**Affiliations:** 1 Department of Financial Markets and Public Finance, Institute of Economics and Finance, College of Social Sciences, University of Rzeszow, Ćwiklińskiej 2, 35-601 Rzeszów, Poland; jzurek@ur.edu.pl; 2 Department of Agricultural Processing and Commodity Science, Institute of Food and Nutrition Technology, College of Natural Sciences, University of Rzeszow, Zelwerowicza 4, 35-601 Rzeszów, Poland

**Keywords:** food purchasing decision, consumer behavior, food choice, sociodemographic factors, COVID-19 pandemic, meat sector, pork, beef, chicken

## Abstract

The coronavirus has wreaked havoc on the global economy before the eyes of the entire world. Due to evolving consumer needs and expectations during the pandemic, the supply and demand for various goods and services varied from the pre-COVID-19 period. This article aims to understand the changes in purchasing and food choices, focusing particularly on meat and meat products, made by consumers and households in response to the crisis caused by the COVID-19 pandemic. The study also indicates the impact directions of these changes and assesses the magnitude of the contribution of various determinants that influenced them. The literature review from 2020 to 2023 was conducted using Scopus and the Web of Science scientific databases. The study identified sociodemographic and individual factors as the main determinants influencing consumers’ purchasing or eating behavior. Positive shifts (e.g., implementing strategies to better manage food at home through activities like creating shopping lists, the average increase in consumer spending during store visits, and decrease in visit frequencies) or negative changes (e.g., shortages of food products in stores due to consumer panic buying, unusually high demand resulting from stockpiling, purchasing fewer fresh products, increased consumption of unhealthy foods and snacking, among other factors) during isolation were influenced by various individual factors (e.g., motivation, mental state) or sociodemographic factors (e.g., gender, age, income level, education). While individual factors had a greater impact on changes in consumer behavior in the early stages of the COVID-19 pandemic, socio-demographic factors became more important as the pandemic progressed.

## 1. Introduction

In March 2020, the World Health Organization declared a global COVID-19 pandemic caused by the SARS-CoV-2 coronavirus [1]. The emergence of the virus prompted countries worldwide to implement diverse levels of preventive measures and restrictions to curb its spread [2,3]. With no specialized treatment or vaccination available, one-third of the world’s population was advised to stay at home to limit the infection’s spread [4]. The restrictions related to the COVID-19 pandemic undeniably impacted the entire economy and society, leading to direct effects on production, supply chain instability, and financial markets. Consequently, numerous economic ramifications spread across various industries, including agriculture, manufacturing, the food market, oil, finance, etc. [5,6].

The pandemic had a tremendous impact on food supply and production, resulting in disruptions [5]. Faced with isolation, changes in behavioral patterns and habits were observed among specific social groups [4]. Understanding the experiences of consumers and households becomes particularly crucial in terms of changes in their purchasing decisions and food consumption patterns in response to the COVID-19 pandemic [7]. This presented challenges to consumers, including the possibility of job loss, uncertainty about the future, and lower incomes, potentially leading to spending reductions, including on food [8].

The pandemic had a significant impact on the global economy and the food supply chain, which is a large sector of the economy, was struggling. Particular attention was paid to the meat and meat products market because every part of the supply chain, starting from the farm, was closely interconnected. Uncertainty has arisen: will consumer demand for meat and meat products remain at the same level, or will it increase or decline? The coronavirus spread to workers, which in turn triggered a wave of closures or reduced speeds of production lines, resulting in a lower supply of meat in stores and pressure on producers. In the case of households, this resulted in partial or total loss of income. Increased prices of selected food items have affected consumer behavior in the food market. In addition, the closure of many businesses and restaurants has increased the demand for home-cooked food. The information collected can be used to develop procedures to mitigate the effects of future crises (if they occur).

Positive shifts (e.g., implementing strategies to better manage food at home through activities like creating shopping lists and increasing home food preparation) or negative changes (e.g., shortages of food products in stores due to consumer panic buying, unusually high demand resulting from stockpiling, snacking, among other factors) during isolation were influenced by various individual factors (e.g., motivation, mental state) or sociodemographic factors (e.g., gender, age, socioeconomic group, income level) [9,10,11,12,13,14,15,16]. The COVID-19 pandemic impacted all facets of the population’s daily life, encompassing consumers’ shopping and eating behavior, food consumption, as well as perceptions of food safety [17].

In this review, our primary aim is to comprehensively explore the changes in purchasing and food choices, focusing particularly on meat and meat products, made by consumers and households in response to the crisis caused by the COVID-19 pandemic. The study also indicates the impact directions of these changes and assesses the magnitude of the contribution of various determinants that influenced them. Most of the available papers discuss only a specific section on changes in consumer behavior during the crisis. In our literature review, we comprehensively discuss changes in consumer income and expenditures, as well as the impact of selected determinants on changes in consumer purchasing and eating behavior in various countries around the world during the COVID-19 pandemic with a particular focus on the meat and meat products market. An analysis of existing publications reveals the global effect of the pandemic in the study area. This paper aims to deepen and summarize the current knowledge of changes in consumer behavior during the crisis caused by the SARS-CoV-2 coronavirus. We discuss the benefits and negative effects resulting from changes in consumer behavior in the food market during the pandemic. Our review also provides valuable information on changing consumer behavior in the food market, among other things, in order to gain a broader understanding of their relevance to science, as well as to provide valuable insights that can help to better understand these mechanisms.

## 2. Methodology

The study consisted of a collective analysis of the results of many individual papers that had been previously described in original publications devoted to specific topics. The authors collectively analyze the results of many articles, which leads to the synthesis of the results of individual original studies into a whole and the alignment of emerging trends and dependencies, thus shedding new light on the examined issues and leading to conclusions that are the results of the source articles. A significant advantage of this review of individual studies is that it offers the opportunity to increase the precision and accuracy of results by combining data from several smaller primary studies. This combination results, for example, in increasing the size of the study sample, which increases the credibility of the conclusions drawn.

The paper raises a research question—how has the COVID-19 pandemic influenced consumer purchasing and consumption changes in the food market in selected countries, with a focus on meat and meat products, and which factors determined them and in what direction?

A comprehensive literature search was conducted in November/December 2023, utilizing Scopus and Web of Science databases. Scopus, recognized as the world’s leading and rapidly expanding bibliometric database, boasts over 94 million records [18,19]. Web of Science (Clarivate Analytics) offers access to numerous databases, including more than 34,000 peer-reviewed journals, 134,000 books, and 300,000 conference proceedings [20].

The primary aim was to provide a comprehensive overview of the available evidence related to changes in consumer behavior and habits in the food market during the COVID-19 period. Therefore, combinations of keywords, including COVID-19, consumer behavior, meat sector, pork, beef and chicken were used to identify the most relevant studies. The first search criterion (COVID-19 and Consumer behavior) yielded 2762 bibliometric records in Scopus, including 2096 articles and 94 reviews. Meanwhile, the criterion of COVID-19 and Meat sector produced 99 records, including 70 articles and 19 review articles. In the Web of Science sampling process, the records for both criteria were 2445 and 82 results, including 2269 and 70 articles, and 96 and 11 review articles, respectively. Subsequent search criteria included the keywords COVID-19 and Pork, COVID-19 and Beef, and COVID-19 and Chicken. To synthesize knowledge, a search strategy was developed. The subjective scope of the study includes consumers, and the objective scope includes their behavior in the food market and the determinants that shape this behavior. The authors classified the factors influencing consumer behavior as follows: individual, psychological and sociodemographic. After conducting searches in both databases, duplicate entries were automatically eliminated. The title, abstract and keywords of each paper were thoroughly investigated by the researchers to find suitable studies as per the scope of the research. For further analysis, 346 articles most relevant to the topic and purpose of the paper were included. After selecting the articles, only those research studies were included in the analysis, which provided information that the research was based on a representative sample. In the case of review articles, papers that assessed the causal impact of the COVID-19 pandemic on the purchasing and eating behavior of consumers in the food market and the causal impact of the pandemic on the meat market were considered.

The specific criteria for including articles in this review were as follows: studies involving consumers in the food market, the COVID-19 crisis, and the meat market during the pandemic, and full-text articles and papers published in 2020–2023. Research and review articles were considered for inclusion. The exclusion criteria were studies from before 2020 and those assessing consumer behavior and the meat market during crises other than COVID-19. Based on the results of reviews and original studies of selected works, the positive or negative impact of a given factor was determined. However, such a state was observed only when an effect or connection in the same direction was found in two-thirds or more of the included papers. The results of this analysis are summarized in Table 1. When the results of research articles were analyzed, Table 2 included only the data that were based on research carried out on a representative sample or the causal impact of a given factor was significant.

## 3. Changes in Consumer Buying Behavior in the Face of the COVID-19 Crisis

The COVID-19 pandemic significantly influenced consumer buying behavior. Table 1 presents data regarding the impact of COVID-19 on consumers’ eating and purchasing behavior, as well as changes in their income and expenditures. These changes have been linked to factors such as the closure of schools, businesses, and restaurants, and restrictions on outdoor activities and socializing [17]. Due to evolving consumer needs and expectations during the pandemic, the supply and demand for various goods and services varied from the pre-COVID-19 period [36]. Studies by multiple authors have revealed disruptions in food supply chains, restrictions on exports and imports, food price inflation, job losses, uncertainty about the future, and limited access to supermarkets. These events have impacted shifts in consumer attitudes and behavior [6,17,37,59,60,61]. 

### 3.1. Changes in Consumer Income and Expenditures

Panzone et al. [36], in a study measuring the impact of a pandemic shock on the sales of UK food retailers and restaurants during the lockdown period, discovered an excess of 5–10% in sales among food retailers. This outcome aligns with significant stockpiling by consumers. Grocery stores and nonstore retailers reported sales increases exceeding GBP 4 billion in the March–August 2020 period compared to the corresponding period in 2019. In contrast, restaurants reported substantial revenue losses [36]. In a study conducted in Spain during the lockdown period, Vall Castelló and Casasnovas [38] observed sales of 12 different food products across three age groups of the population. The authors identified an increase in average weekly sales of about 2.93% compared to 2019. The highest rate of purchase levels was observed among those aged 18–35 (4.13%), followed by those aged 36–65 (3.42%), and 66 years+ (1.16%). In the oldest group (66 years+), the shopping pattern remained almost identical in 2019 and 2020 for all weeks [38]. Concurrently, Bakalis et al. [21] highlighted a clear change in consumer behavior or purchasing habits in the United States. During each store visit, there was an average increase in consumer expenditures of about 15%, while the number of visits decreased. This change may have been influenced by the preparation of meals at home for the entire family. Drummond and Hasnine [62] found that higher-income individuals were less likely to buy in stores compared to lower-income individuals, who were more likely to travel by bus or subway [62].

COVID-19 has diminished the frequency of shopping, driven by uncertainties regarding mobility and personal health limitations. Additionally, nonmonetary costs of shopping, including perceived risks, queues, and the use of masks, have increased [36]. Practices associated with changing shopping habits, such as reduced outings but with higher expenditures or adjustments to shopping hours, typically necessitated access to a car or reliable public transportation. For some people, the act of going out shopping became challenging or impossible due to recommendations for isolation and/or staying at home. For those with lower incomes, local shopping became a necessity rather than a choice due to the lack of personal means of transportation (e.g., a car) to reach large supermarkets often situated outside the city [10]. The limited access to daily shopping may lead to a decrease in the consumption of fresh foods, particularly fish, vegetables, and fruits, in favor of highly processed foods like ready-to-eat snacks, convenience foods, and junk foods, which often contain high levels of sugars, salt, and fat [63].

The COVID-19 coronavirus has wreaked havoc on the global economy before the eyes of the entire world. The virus not only impacted business operations but also compelled the closure of numerous establishments due to social distancing and lockdown measures [64,65,66,67]. Concerning employees, this led to psychological challenges stemming from job loss, reduced income, and uncertainty about the future [67]. The rise in food prices during a period of declining incomes poses a significant problem for consumers [68]. Elleby et al. [68] discovered that the overall loss of society’s income influences changes in consumer expenditures.

Low-income consumers typically allocate a major portion of their income to food consumption [69]. Fluctuations in food consumption primarily result from the substitution of grains and vegetables for higher-end products [70]. For higher-income consumers, the percentage of income spent on food consumption drops significantly, diminishing the impact of income growth on food demand. The demand for food shifts towards “convenience and quality factors” for higher-income individuals, emphasizing earlier preparation and consumption away from home, rather than an overall increase in food consumption [70,71].

In a questionnaire-based study, Dou et al. [23] examined the impact of the pandemic on food-related issues in 1547 households in the United States and 1732 households in China. The researchers discovered that 25.6% of US respondents reported significantly higher prices for fish, meat, and eggs, while Chinese respondents reported notably higher prices for nearly all food items. Approximately 50% of the Chinese cohort reported significantly higher prices, primarily for fish, meat, and eggs. According to the survey, the authors also determined that 62.4% of Chinese respondents acknowledged the presence of a household member(s) who experienced income loss due to the pandemic. In comparison, in the US, the percentage was 35.7% [23].

Chronopoulos et al. [25], analyzing consumer spending responses to the emergence and spread of a pandemic in the UK (England, Scotland, Wales), discovered that in the two weeks following the World Health Organization’s declaration of COVID-19 as a pandemic, there was a significant surge in spending on groceries consistent with panic buying and stockpiling. As the government-imposed lockdown loomed, discretionary spending (defined as the sum of spending in categories such as groceries, alcohol, drinking and eating, gambling, gaming, and other related items that individuals can directly influence) sharply declined, and this downward trend persisted throughout the lockdown period. At the same time, the authors observed variations in spending levels among countries. Consumer spending on groceries in Scotland remained significantly elevated throughout the lockdown and sustained that level even after the stay-at-home alert was issued. The authors also incorporated demographic data such as age, gender, and income level in their study. Based on this, they found that during COVID-19, men, older individuals, and those with higher incomes spent more on food expenditures than women, younger people, and those with lower incomes. Chronopoulos et al. [25] affirmed that consumers respond to negative shocks by reducing discretionary spending. Previous evidence suggests that such declines occur due to increased uncertainty, financial constraints, or declining consumer expectations about future financial prospects. Nevertheless, in product categories such as grocery spending, very strong increases in spending were observed as the incidence of COVID-19 infection increased and the inevitability of government-imposed lockdown [25].

Changes in consumption leading up to the lockdown became evident through variations in transaction volume. In March 2020, compared to the previous year in New Zealand, the number of transactions declined in most types of retail, while the value of transactions remained relatively unchanged. The period before the lockdown coincided with a shift in the value of spending transactions. Average spending per transaction increased by 12.7% before the quarantine in response to the government’s announcement confirming the lockdown. The decrease in the number of transactions, coupled with an increase in the value of transactions, suggests that consumers opted for bulk purchases, reducing the need for more frequent buying. This resulted in higher average spending per transaction, including on groceries and alcohol, compared to the period before the lockdown. A significant decline in consumer spending due to the lockdown occurred in the hospitality sector [22].

Bangladesh, following India, became the second most pandemic-affected country in South Asia. The COVID-19 crisis led to fluctuations in supply and demand, adversely impacting food affordability. Retail stores experienced shortages of goods, contributing to consumer stress. Workers faced the challenge of irregular wages and job losses during the crisis. To cope with rising food prices, impoverished households turned to consuming less nutritious and cheaper food. Among demographic factors such as income, age, and gender, only income played a significant role. A rise in income category correlated with more informed consumer behavior, a decrease in the risk of food insecurity, and reduced exposure to higher food prices [5]. Rabbi et al. [5] demonstrated that income was the most influential factor shaping changes in consumer behavior, followed by food prices. It is noteworthy that even before the COVID-19 crisis, developing and impoverished countries like Bangladesh dealt with unstable supply chains, exacerbating minor food security risks [5]. Similar findings were observed by Adeeth Cariappa et al. [72], who investigated the effects of lockdown in India. The authors confirmed that the pandemic triggered significant shifts in food prices and unprecedented panic buying. A majority of consumers (75.31%) were affected by the price increase during COVID-19. Additionally, 92% of respondents reported changes in shopping and consumption behavior, particularly those experiencing negative changes in income due to reductions or layoffs [72]. Consumer income emerged as the most critical determinant of consumption behavior [71,73,74]. In March 2020, Wolfson and Leung [26] conducted a survey in the United States on the impact of COVID-19 on low-income adults. The authors affirmed that the crisis exacerbated existing disparities, negatively impacting low-income households struggling to meet basic needs and facing food security challenges [26].

In a study on food stockpiling during COVID-19 in China, Wang et al. [37] revealed that women, people with higher education, and consumers with high incomes were more likely to stockpile food on a larger scale. However, the willingness to pay (WTP) for fresh food reserves depended solely on the income level. Additionally, women exhibited greater risk aversion compared to men [37].

Household income has a direct association with the level and composition of consumption spending. Income losses and disruptions to local supply chains due to COVID-19 have undoubtedly led to an increase in food insecurity in many developing countries. Uncertainty regarding the flow of income during the pandemic was very high, especially in developing and impoverished countries, such as, e.g., Bangladesh or India. Most vulnerable workers lost their jobs overnight due to the lockdown, and when it was lifted, their jobs were only partially restored due to the paralysis of businesses. The pandemic has had the most serious impact on food availability in less economically developed countries. Its effects were felt not only in disruptions to availability but also in a shift in consumer demand towards less nutritious, cheaper foods due to declines or loss of income. Household food security in these countries has been disrupted to a much greater extent than in highly developed countries, where employment was more stable, which in turn was associated with a lower risk of loss of income in the household. For example, in the United States, food insecurity only affected a percentage of households with the lowest incomes.

Large households are at greater risk of food insecurity compared to small and medium-sized households due to the additional burden of food consumption. Income-driven declines in food consumption have only exacerbated the prevalence of malnutrition in underdeveloped and developing countries.

### 3.2. Influence of Psychological and Individual Factors on Changes in Consumer Shopping and Eating Behavior

Psychological factors are believed to have played a pivotal role in influencing consumer anxiety and panic buying behavior following the COVID-19 outbreak [61,75]. The pandemic disrupted both the demand and supply sides of consumption, leading to disruptions in the supply of products and services. Simultaneously, it impacted the volume of consumption and product purchases [76]. Food supply disruptions were linked to factors such as consumer panic buying, a radical shift in consumption patterns from food service to home cooking, labor shortages, and transportation network failures due to national issues and border controls [39]. Supply chain disruptions and panic buying can limit access to fresh food, tipping the balance toward greater availability and consumption of highly processed foods with longer shelf lives [77].

Abnormally high demand leads to elevated inflation rates, increased prices, and the potential imposition of purchase quotas. Panic buying, in turn, has a significant impact on consumer anxiety about food shortages [6,59,60,61,78,79]. Studies indicate that a negative mood and herd psychology contribute to panic buying. The herd effect (imitation effect) is defined as being influenced by others—the tendency of consumers to buy a particular good simply because others are also purchasing it. Such individuals express a desire to align their behavior with the majority of market participants [80].

The sudden restriction of international and domestic mobility has resulted in labor shortages in countries dependent on seasonal migrant workers in the agri-food sector, affecting food availability and prices worldwide [81,82]. While certain parts of the supply chains were functioning normally, panic buying itself caused shortages of various products on supermarket shelves due to stockpiling of staple foods, including canned foods, legumes, rice, pasta, and frozen foods [14,17,53,83,84,85,86].

In a survey of US residents, Bender et al. [7] revealed that 27% of respondents expressed fear of running out of groceries when shopping, with 74% indicating concern about raw materials and products such as meat, canned goods, frozen foods, baking items, dairy, pasta, and fresh produce. Brizi and Biragli [4] found that people with a greater need for cognitive closure (i.e., a desire for predictability in times of uncertainty) purchased more food during the pandemic, as they were more likely to perceive having insufficient food in their homes [4].

Deschasaux-Tanguy et al. [27] observed changes in food supply among 37,252 French consumers during the COVID-19 period, which resulted in a decrease in the purchase of fresh produce (27.4%) and difficulties accessing preferred products (13.7%) and organic foods (12.3%). The reduction in buying fresh produce stemmed from consumers engaging in less frequent grocery shopping and/or facing challenges in accessing their regular grocery stores or preferred products [27].

The closure of food service and limited-service restaurants has impacted consumers’ purchasing and eating habits, leading to a shift in demand from food service to retail [87]. Dou et al. [23] demonstrated during the COVID-19 pandemic that in China and the United States, the frequency of ordering food from take-out or delivery restaurants declined. At the same time, 74% of all respondents reported going out for grocery shopping less frequently during the pandemic than before. Online grocery shopping with curbside pickup or home delivery gained popularity. This research aligns with findings from Bender et al. [7], who revealed that over 25% of surveyed US residents utilized grocery pickup or delivery services. Grashuis, Skevas, and Segovia [88] found that the number of new COVID-19 cases influences grocery shopping preferences. For example, consumers in communities with an increasing rate of coronavirus spread were less likely to make grocery purchases at stationery stores, while in communities with a decreasing virus spread, the preference for home delivery over other available methods was weaker [88].

Samuk and Sidorowicz [89] showed that 46% of university students in Poland altered their previous shopping behavior. Respondents primarily indicated a decrease in the frequency of shopping at brick-and-mortar stores; following the pandemic outbreak, they were less likely to visit discount stores (69%) and big-box stores (76%). At the same time, 90% of students acknowledged that during the pandemic, they increased the frequency of online shopping. This shift may have resulted from the fear of leaving home and the mental comfort associated with avoiding the risk of infection for themselves and their families. At the same time, respondents mentioned that groceries were not the category of products most frequently purchased online (13%), potentially influenced by the fact that the surveyed group consisted of young people [89].

Werner-Lewandowska et al. [24] showed a reduction in the frequency of grocery shopping among 214 respondents in Poland while buying more groceries per shopping trip. Laguna et al. [90] demonstrated that respondents from Spain mainly reduced shopping trips to once a week (76.5%) or twice a week (13.65%), with daily shopping nearly disappearing (1.12%). Comparing the precrisis period, consumers’ shopping frequency decreased to twice a week (50%) or one outing a week (35%) [24].

Among various behavioral factors believed to underlie panic buying decisions, the perceived importance of panic buying was most strongly correlated with the need for control, the desire to minimize the number of trips to grocery stores, as well as the belief that it was a smart move [12].

Eating habits undergo modifications under stress [91]. Negative emotions can impact food intake as much as positive emotions, with numerous studies indicating that individuals tend to consume more high-energy foods when faced with negative emotions. Constant exposure to pandemic-related news in the media can induce stress, leading to overeating, especially of highly palatable, sugar-rich foods [63,92,93,94,95]. Research affirms that the sweet taste or sugar can alleviate stress by inhibiting cortisol secretion or activating endogenous cannabinoid receptors [96,97,98]. Feelings of boredom resulting from prolonged periods at home may be linked to overeating as a means of escaping monotony [94,99]. The condition where a person cannot distinguish between hunger and emotional arousal is referred to as emotional eating, characterized by the tendency to eat in response to negative emotions, with the chosen foods being primarily energetic and palatable. Higher emotional motivation in situations of depression, stress, and boredom can result in eating as a form of emotional comfort [94]. Conversely, consuming carbohydrate-rich foods can alleviate stress by increasing serotonin levels, positively affecting mood [100,101].

Evidence points to profound psychological and social effects related to anxiety, fear, depression, fear of contagion, and insomnia among the public arising from the coronavirus disease pandemic. The psychological aftermath of the pandemic is likely to persist for months and years [102,103]. Deschasaux-Tanguy et al. [27] observed emotional reasons for food consumption among French consumers during the COVID-19 pandemic such as eating out of boredom or anxiety. A study by Bemanian et al. [28] conducted in Norway among 24,968 adults also found emotional eating in 54% of respondents, which was observed much more frequently in women. Risk factors for the development of this condition were concerns related to health, personal finances, and mental stress [28]. Moreover, emotional eating was observed in 64% of participants in the Ecuadorian study, and its frequency increased with the severity of perceived stress associated with the COVID-19 pandemic. Women reported more perceived stress than men (77.91% and 22.09%, respectively) [29]. Coulthard et al. [30] examined changes in eating patterns and behaviors during isolation in the UK among 620 participants. The authors found that an increase in the consumption of energy-dense snacks during isolation was associated with prequarantine eating behaviors (uncontrolled and emotional eating) and COVID-specific health anxiety. At the same time, Scarmozzino and Visioli [8], in a survey conducted in Italy, showed that almost half of the respondents (49.6%) did not significantly modify their diet during isolation, but 52.9% reported consuming more food. The consumption of ice cream, desserts, and chocolate (42.5%) and salty snacks (23.5%) increased due to higher levels of anxiety reported by respondents (42.7%). Dou et al. [23] also confirmed that consumers from Chinese and US households found comfort in food during COVID-19, with about 70% of all respondents indicating that food was an element that influenced stress reduction and coping with boredom during COVID-19. In addition, food was an element of comfort and pleasure [23].

Evidence also suggests that the implementation of lockdown and social distancing measures resulted in changes in consumer habits that adversely affected health, including prolonged TV viewing and increased snacking, elevated consumption of processed foods, and reduced physical activity. Individuals faced with COVID-19 had limited access to healthy foods, ate more frequently even when not hungry, and were less physically active [31,34,104,105,106]. The primary factors contributing to weight gain during the pandemic were reduced physical activity and increased food consumption [32]. Deschasaux-Tanguy et al. [27] observed unfavorable trends among French consumers in March–May 2020, including a decline in the consumption of fresh foods, particularly fruits (17%) and fish (31%), and an increase in the consumption of cakes and pastries (20%) as well as chocolate and sweets (22%). At the same time, 22% of respondents reduced their intake of fresh red meat. Respondents experienced a decrease in physical activity (53% of respondents) and an increase in sedentary lifestyles (63% of respondents). Furthermore, 56% of respondents admitted to modifying their dietary practices during isolation, primarily due to lifestyle changes—altered routines, spending more time cooking meals, refraining from eating out, and difficulties in maintaining a regular meal schedule [27].

During the pandemic period, approximately 40% of study participants residing in the United States reported weight gain, while 18.2% experienced weight loss. Those who gained weight exhibited eating behaviors such as increased snacking (*p* < 0.001), frequent consumption of ultra-processed foods (*p* < 0.01), lower levels of physical activity, reduced hunger control during peak lockdown, and high stress levels [33]. Similarly, in a survey conducted by Dou et al. [23], the proportion of respondents reporting weight gain outnumbered those reporting weight loss [23].

### 3.3. Influence of Sociodemographic Factors on Consumer Eating and Shopping Behavior

Ignoring sociodemographic factors, such as gender, education, age, employment status, and household composition, makes it challenging to comprehend how the COVID-19 pandemic impacted consumer demand for foodstuffs [14]. It can be inferred that irrespective of the product category, consumer behavior has been altered [107].

Isolation resulted in an increase in time spent at home, providing consumers an opportunity to modify their cooking habits. Prior to the COVID-19 pandemic, there was a consistent decline in household food preparation [108,109,110]. During the lockdown, with the closure of most food outlets, the public shifted towards preparing meals at home [109]. For instance, Bedner et al. [7] observed an increase in home food preparation reported by 62% of US respondents compared to the pre-pandemic period. Similar findings were reported by Altarrah et al. [17], confirming that 76% of Kuwaiti respondents noted an increased frequency of eating at home. Consequently, consumers took actions to better manage food at home, such as creating shopping lists, preparing meals creatively, freezing food more often, and depleting supplies [17,111]. Household size showed a positive correlation with the frequency of cooking at home [7]. In contrast, changes in cooking were not differentiated by gender, place of residence, education level, occupational status, or age [112].

Górnicka et al. [34], in a survey of Polish food buyers, identified two consumer patterns characterizing dietary changes—prohealthy and unhealthy. Adherence to the prohealthy pattern was negatively associated with age but positively linked to being overweight or obese before the pandemic. It was revealed that adults over 40, the unemployed, those living with children, residents of regions with higher GDP, and those not consuming home-cooked meals may be more likely to adopt unhealthy behaviors. During the pandemic, one-fifth of the prohealthy pattern sample increased their intake of low-fat meat and/or eggs, legumes, fruits, and whole grain products. These respondents concurrently demonstrated increased consumption of vegetables, milk and dairy products (around 30%), and water (approximately 50%). In this pattern, 50% of respondents reduced their intake of salty snacks and sweets. The unhealthy pattern was characterized by an increased consumption of fast food, ice cream, and processed meat (about 20%), salty snacks (50%), homemade cakes (70%), and confectionery (80%). In addition, 60% of those surveyed in this pattern reduced their consumption of fruits and vegetables, 30% of fish, and 25% of whole grain products. In both patterns, the percentage of respondents eating home-cooked meals increased (>50%) [34].

Deschasaux-Tanguy et al. [27] categorized 37,252 French consumers who completed lockdown questionnaires in April–May 2020 into three distinct participant groups. Nutrition-related changes and their sociodemographic, health status, and lifestyle correlates were examined using multivariable logistic regression models, defining clusters through an ascending hierarchical classification of change profiles. Cluster one, comprising 42.9% of participants, represented individuals who maintained a stable diet, weight, and physical activity practices during the pandemic. This cluster was associated with male gender, older age, lower education level, normal weight, smoking, having a job outside the home during isolation or inactivity before isolation (e.g., unemployed, retired), being in a relationship, living in rural areas or cities with fewer than 100,000 residents, having lower levels of anxiety and/or depressive symptoms, and maintaining a higher quality diet before the lockdown. Individuals in this profile likely had established habits or were less disturbed by the environment/lifestyle during the COVID-19 lockdown. Cluster two, encompassing 37.4% of participants, included individuals who experienced adverse changes in consumption during isolation. These changes involved problems with maintaining a regular meal schedule, buying fewer fresh products, eating more often out of boredom and/or due to anxiety, and snacking more frequently than once a day. Respondents in this cluster reported an increase in consumption of cakes/cookies, chocolate/sweets, savory pates/sandwiches/pizza, potatoes, cheese, and alcoholic beverages, along with a noticeable decrease in the consumption of fresh products (meat, fruits, and vegetables). They also reported spending more time preparing home meals. This pattern was associated with female gender, younger age, remote work, and having children at home, indicating parents caring for their children while maintaining their work activity. This pattern was also associated with greater consumption of ultra-processed foods before the lockdown, higher levels of anxiety and depressive symptoms, and lower income. This profile suggested fewer opportunities (e.g., technical, financial) to engage in health behaviors and may explain observed behaviors such as snacking and the consumption of comfort foods like cookies and sweets. Cluster three, constituting 19.8% of respondents, reported favorable changes related to nutrition during isolation. These changes included reduced consumption of sweets/chocolate and cookies/cakes, increased consumption of fresh fish, vegetables, and fruits, a desire to balance their diet, and spending more time cooking home meals than before the pandemic. This cluster was associated with younger age, higher levels of education and income, being overweight/obese, smoking, being partially/technically unemployed, being a student or working from home while isolated, not having children under 18 in the household, experiencing more anxiety but fewer depressive symptoms, and typically having a lower usual diet quality. People in this cluster were also less likely to have children under 18 in their household than cluster two [27].

Rodrigues et al. [35], in a survey conducted in May 2020 during the pandemic, assessed Brazilians’ food habits and perceptions, emphasizing changes in food consumption and choice. Respondents reported increased food buying and consumption, with women predominantly maintaining a less healthy diet. About 81% of participants noted changes in their eating habits during the quarantine period. Approximately 60.48% observed an overall increase in the amount of food consumed, while 51.93% partially or fully agreed that food consumption had risen during quarantine. About 36.5% of respondents indicated buying more food and shifting to different previously purchased and consumed food categories (46.01%). Notably, women were more likely than men to increase their intake of less healthy foods, particularly sweets, a trend potentially linked to increased time spent at home and emotional states [35]. At the same time, Altarrah et al. [17], in a study conducted in Kuwait, revealed that 33.7% of respondents changed their eating habits during the pandemic. Participants cited isolation, sedentary lifestyles, television viewing, and the use of electronic devices as reasons for increased food intake. The study found statistically significant differences in eating habits between genders. More men reported consuming less food, with the stated reason being the reduced availability of food at home compared to women. Food purchasing behavior also showed statistical differences between genders, where female respondents shopped less frequently but bought more food items each time, including prepared frozen foods. On the other hand, men purchased statistically significantly more sweets, salty snacks, and canned goods, and were more often concerned about food supply [17].

The study by Ogundijo et al. [113] provides empirical evidence of the pandemic’s impact on the eating and purchasing behaviors of individuals in England, assessed through sociodemographic variables. COVID-19 significantly influenced the healthier food purchasing decisions of respondents aged 23–38, with participants in the 55–73 and 74–91 age groups being the least affected. The impact of the pandemic on purchasing healthier food was more pronounced for younger generations and those employed part-time or full-time compared to retired respondents over the age of 55. There was a notable decrease in the amount of food purchased with increasing age. Working individuals and students experienced a significant impact on the amount of food purchased during the pandemic. The level of education exerted the most substantial influence on the eating behavior of participants with higher education levels, possibly because these respondents were more health-conscious and inclined to buy healthier foods [113]. Bolek [40] conducted a questionnaire-based study from March to December 2020 among 992 Turkish consumers, confirming the pandemic’s impact on changes in eating and food purchasing habits (*p* < 0.05). Approximately 65% of respondents aimed to consume more immune-boosting foods, while 58% were more inclined to purchase fresh products, irrespective of age. Since the onset of the pandemic, 64% of respondents attempted to consume more fruits and vegetables, with consumers over the age of 65 displaying a greater emphasis on healthy eating compared to other age groups (*p* < 0.05), possibly due to the higher risk of complications from COVID-19. Food preservation practices, such as freezing, allow for fewer shopping trips, with 66% of consumers adopting this approach during the pandemic (*p* < 0.05) [40].

Changes in food supply channels played a pivotal role in altering consumer purchasing behavior amid the challenges posed by the COVID-19 crisis. Throughout the pandemic, consumers adopted new habits or modified existing ones [74,86,114,115]. Altarrah et al. [17] conducted a survey of consumers in Kuwait during the pandemic and discovered that 71.2% of respondents expressed fears of contracting the virus during in-person food purchases. Notably, more women in comparison to men took precautions during in-person purchases, as evidenced by more frequent hand disinfection and the use of credit card payments [17]. The pandemic witnessed an increase in the availability of online stores, aligning with shifts in consumption patterns [116]. Gender emerged as a highly influential factor (*p*-value < 0.01) in shaping behaviors and habits, such as the use of delivery apps and ordering take-out or fast-food meals with delivery [117]. Consumers across all demographic groups, particularly those under 65, initiated online food purchases (*p* < 0.05) [40]. Hassen et al. [117] surveyed 579 respondents in Qatar and found that younger participants were more inclined to order groceries online compared to their older respondents. In contrast, respondents with higher education levels were more likely to engage in online grocery shopping and tended to dine with family members more frequently than other respondents [117]. Women exhibited a propensity for more online purchases compared to their male counterparts [116]. Truong and Truong [118], in a study involving US residents, observed that people living apart were more inclined to use curbside pick-up or spend more on in-store shopping than married respondents. This inclination might be attributed to the preference of separated individuals to visit stores, potentially seeking more contact with the public [118].

## 4. Meat and Meat Products Market in the COVID-19 Period

In a short period, the SARS-CoV-2 virus brought the economies and social activities of the affected countries to a standstill. The disruption of supply chains significantly complicated international trade, prompting meat processing plants to swiftly adapt to the new circumstances [43]. Table 2 presents impact of the COVID-19 pandemic on price changes, fluctuations in consumption and purchase of different meat types in selected countries.Ensuring the safety and uninterrupted continuity of production became paramount for processing companies, leading to the implementation of enhanced measures to safeguard the health of workers [43]. The global impact of the COVID-19 pandemic extended to food safety considerations worldwide [119]. Meat, being a focal point in food safety discussions, gained prominence, particularly given the influential factors of rising global incomes and the expansion of the middle class in emerging countries, contributing to increased demand for food, including meat [120,121]. In the United States, COVID-19 has reportedly affected at least 93 farms and production facilities, 257 food processing plants, and 462 meat-packing plants. At least 54,036 workers were identified as infected with the SARS-CoV-2 coronavirus, with a significant portion, around 39,905, involved in packing [87]. Due to the slowdown in production lines, the number of animals slaughtered decreased and, consequently, there was a decrease in the quantity of the product, accompanied by an increase in demand [122]. In response to 246 positive cases in England and Wales, several meat-packing plants temporarily halted operations [87].

The retail prices of all meat types in Bangladesh experienced an upswing during the COVID-19 pandemic. This surge was attributed to heightened costs associated with animal purchases, disruptions in the supply chain, labor shortages, and increased labor costs in slaughterhouses [123]. A major meat processing company in Bangladesh reported a 20% decline in mutton and beef sales to retail customers and a substantial 80–90% decline in business-to-business sales. This decline was mainly due to a reduction in restaurant and hotel operations, as well as the reduction in social ceremonies during the pandemic. Concurrently, the company witnessed a production decrease of approximately 30–40%, coupled with rising production costs. These cost increases were linked to measures implemented to ensure the safety and housing of workers, as well as the sourcing of spices and other raw materials crucial to the production process. Another significant meat processor noted a surge in demand for frozen chicken in supermarkets by around 10–15%, while franchise stores experienced a decline of at least 60% in fried chicken sales [123].

Meixner and Katt [120] investigated beef preferences through a consumer survey conducted in the United States (*n* = 999), utilizing a choice-based experiment. Assessing consumers’ valuation of beef attributes, the authors gauged the WTP for this commodity during the COVID-19 period. A negative WTP value was associated with a country of origin other than the US. Notably, significant correlations were identified between statements related to economic and financial situations and respondents’ expectations. For individuals who had recently lost their jobs, the price attribute held greater importance, resulting in a lower overall WTP value for this group. Pessimistic respondents placed higher importance on low prices, concurrently showing greater tolerance for considerations of food safety and country of origin. Personal perceptions of the COVID-19 pandemic’s impact significantly influenced perceptions of beef traits, confirming the hypothesis that those more affected by the pandemic became increasingly price-sensitive. Consumers with higher incomes demonstrated a willingness to spend more to ensure the safety of the beef they purchased. Notably, individuals who recently lost their jobs appeared to be most affected by the COVID-19 pandemic [120].

The beef industry stands as a crucial component of agricultural activities in Colombia [124]. Ramírez et al. [124] found that larger households and those with higher incomes were less likely to reduce their beef consumption compared to single-person households during the pandemic. Conversely, households experiencing declining incomes due to COVID-19 were more inclined to reduce their beef consumption, indicating the substitution of this commodity with cheaper alternatives during isolation. Importantly, the disparity between the high percentage of households facing income reduction during lockdown (83.8%) and the more moderate percentage reducing beef consumption (47.8%) suggests that the consumption of this commodity did not decrease in proportion to declining household income [124].

Récky et al. [52] conducted a survey on changes in purchasing behavior among 565 respondents facing multiple crises, including COVID-19, the war in Ukraine, and high inflation. The findings indicated a notable shift in dietary preferences in Slovakia during the pandemic, with many individuals opting for a plant-based diet over an animal-based one. Sales of plant-based foods, including meat substitutes, witnessed an increase, while sales of animal meat declined by [52]. Celik and Dane [125] demonstrated a shift in food consumption preferences before and during the pandemic across various countries, including Nigeria, Turkey, the USA, and European countries. Before COVID-19, meat and bread were the top preferences, but during the pandemic, fruits and vegetables took precedence. This change could be attributed to the belief that fruits and vegetables contain essential vitamins to combat the virus [125]. In Slovenia, higher levels of COVID-19 risk perception were associated with a decrease in fresh meat consumption, while lower levels of this factor influenced an increased likelihood of bread consumption [53]. The motivation behind increasing the consumption of plant-based products was often driven by the desire to boost immunity and attain other health benefits [52]. Additionally, plant-based foods were considered a more economical dietary alternative to meat, fish, and dairy [52,126]. The literature suggests that a plant-based diet contributes to the proliferation of positive bacteria in the gut, leading to improved immunity and overall health [127]. Mazurkiewicz [128], citing Market Research World, reported that over 40% of Poles altered their eating habits during COVID-19, with one in four individuals reducing their meat intake. Simultaneously, there was an introduction of plant-based meat substitutes into diets and a reduction in dairy consumption. Meat processors keenly observed the rising vegetarian trend, incorporating plant-based alternatives into their commercial offerings [128].

Bolek [40] found that, during the COVID-19 pandemic, 51% of Turkish consumers made no effort to reduce their red meat consumption. No statistically significant differences were observed across age groups (*p* > 0.05). Similar results were obtained by Chlipala and Żbikowska [129] in a survey of 750 respondents in Poland, where 59.7% of participants indicated that they purchased as much meat and cold cuts as before the pandemic. Only 23.3% reported buying less of these products, while 10.3% increased their purchases of meat and cold cuts [129]. In a survey of 1048 South Carolina consumers conducted in November 2020, Richards and Vassalos [46] found that the majority (58%) did not alter their local meat (products that are farm-raised in South Carolina within 200 miles of the customer’s home or produced in North Carolina and Georgia within a short distance from the South Carolina border) consumption habits. However, 21.6% increased their meat consumption, and 20.4% reduced or abandoned meat consumption. Reasons for limiting meat consumption included concerns about leaving home during the pandemic, low availability or absence of local meat products, opting for foreign (nonlocal) and cheaper meat, or having a stock of frozen nonlocal meat [46]. When it came to consuming local meat in restaurants, over half of the respondents (51.7%) indicated that their restaurant purchasing behavior remained unchanged, while 38% limited or completely abandoned their consumption. Reasons cited included avoiding dining out during the pandemic, restaurant closures or restrictions on seating capacity, and attempting to save money by reducing eating out [46].

Upadhyay et al. [130] found a statistically significant reduction in the consumption of fish, chicken and beef in the northeastern region of India during the COVID-19 pandemic. Before the pandemic, these raw materials were purchased by 88%, 89% and 37% of households, respectively, while during the crisis, they were purchased by 84% (fish), 86% (chicken) and 29% (beef) of households. This could be due to the limited availability of imported raw materials, price increases, or consumer anxiety about health risks during the pandemic. In turn, Shen and Zhong [131] showed that in the early stage of the COVID-19 pandemic in 2020 in Wuhan and Nanjing, China, there was an adverse impact on the consumption of food of animal origin among 52.4% of respondents. In terms of obtaining pork, poultry, beef and mutton and aquatic products, the pandemic affected approximately 25.8%, 21.1%, 26.6% and 28.3% of families, respectively. In total, 29.7% of households experienced a decline in income due to the crisis caused by the COVID-19 pandemic. In addition to loss of income, the consumption of food of animal origin was also influenced by price increases and insufficient household savings. Revenues from meat sales have reportedly declined since the onset of the COVID-19 pandemic, primarily attributed to the closure of restaurants. The preference for meat over vegetarian options when dining out has led to reduced demand for meat, particularly impacting the sales of more expensive meat items. Conversely, sales of plant-based meat substitutes in the United States surged by almost 200% in April 2020 compared to the same period in 2018. This increase may be attributed to limited meat availability, heightened food safety concerns, and the competitive marketing of meat alternatives. Producers seized the disruption associated with the COVID-19 crisis as an ideal opportunity to attract new customers [132]. Analyzing Nielsen data, Borsellino et al. [74] reported a 117% increase in sales of ramen, a 126% increase in lasagna and pizza sales, and a 199% increase in pasta sales from the end of January to the end of March 2020 in the United States. During this period, sales of meat products increased by only 31%. The shift toward increased home cooking resulted in higher sales of meat, particularly poultry, pork, and beef. Sausages, hot dogs, and bacon sales increased by approximately 100%, while vegetable meat substitutes, both fresh and frozen, saw a growth of about 70% [74]. In China, Li et al. [133] observed that meat, rice, and vegetables experienced the highest demand during the early stages of the developing pandemic.

During the initial phase of the pandemic, disruptions in meat packing, processing, and distribution capacities led to reduced prices of livestock, particularly poultry, beef, and pork, in the US and European Union member states [43,74]. This, in turn, resulted in a diminished supply and higher prices for meat products at retail outlets for consumers [74].

Poland, as the largest producer of poultry meat in the European Union and a prominent producer of beef and pork, faced significant challenges during the first half of 2020 amid the COVID-19 crisis. The country experienced a decline of 3–5% in beef and poultry exports and a substantial 28% decrease in pork exports compared to the same period in 2019. The meat industry, already grappling with African swine fever and avian influenza, found itself dealing with a compounding set of unfavorable factors, adding to the existing uncertainty about the future. This was particularly critical as over 50% of poultry meat production and approximately 80% of beef production were intended for international markets [43].

Throughout the first and second quarters of 2020, pig slaughters in medium and large companies in Poland recorded declines of 11.5% and 7.5%, respectively, compared to the same quarters in the previous year. This reduction was a consequence of hindered pork exports to foreign markets due to the impact of COVID-19. On the other hand, poultry slaughters increased by 3.5% and 4.4%, while beef livestock levels remained relatively stable. The collapse of meat sales in the hotel and catering segments, a result of the COVID-19 crisis, led to challenges for meat plants in selling high-quality raw materials to regular customers [43]. During the first half of 2020, retail prices for meat and meat products rose by 11.3% compared to the first half of 2019. Pork prices increased by 17.4%, poultry prices by 3.1%, and beef prices by 2.4% [43,134].

## 5. Conclusions

Several studies by various authors confirm the outbreak of the COVID-19 pandemic and the associated freezing of economies, reduced incomes or job losses, and security concerns have decisively left their mark on the purchasing and eating behavior of consumers around the world. The food security of households in developing and impoverished countries, such as e.g., Bangladesh or India, was disrupted to a much greater extent than in highly developed countries (e.g., Western Europe), where employment was more stable, which, in turn, was associated with less risk of loss of income in the household. For example, in the United States, food insecurity only affected a percentage of households with the lowest incomes. Income-driven declines in food consumption have only exacerbated the prevalence of malnutrition in underdeveloped and developing countries. The pandemic exposed vulnerabilities in global food systems, emphasizing the significance of short food supply chains. In less economically developed countries, and even in poorer regions of some countries compared to richer ones, there was a greater increase in the consumption of cheaper meats (e.g., poultry meat) and a greater decrease in the consumption of more expensive meats (red meat). As the pandemic outbreak caused major disruptions in production, market food supply, and consumer incomes, however, the problem was more pronounced in underdeveloped and developing countries. Psychological and emotional reactions to COVID-19 may increase the risk of dysfunctional eating behavior.

Positive changes in household dietary dynamics were observed, including strengthened family ties at the table and perhaps better improvements in nutrition due to the increased time spent planning and preparing meals at home. The pandemic also influenced shifts in consumer shopping behavior concerning the frequency of purchases of certain product categories, the place and channels of shopping, and attitudes toward consumption, but particular changes were determined by individual, psychological, and sociodemographic factors. While individual and psychological factors had a greater impact on changes in consumer behavior in the early stages of the COVID-19 pandemic, socio-demographic factors became more important as the pandemic progressed.

## Figures and Tables

**Table 1:** Impact of COVID-19 on consumers’ eating and purchasing behavior, as well as changes in their income and expenditures.

Variable	Country/Region	Impact	References
Changes in consumer income and expenditures	Many countries, New Zealand, China, USA, Poland	+ (A decrease in the frequency of visits to the store (less likely to become infected with SARS-CoV-2 coronavirus))	[21,22,23,24]
	USA, China	− (Decline in food security among the lowest-income families or those whose household members lost income during the pandemic)	[23]
	Great Britain (England, Scotland, Wales)	− (A marked decline in discretionary spending)	[25]
	USA	− (The crisis exacerbated existing disparities, negatively affecting low-income households struggling to meet basic needs and facing food security challenges)	[26]
Changes in consumer eating behavior	France, Norway, Ecuador	− (Emotional reasons for eating, such as eating out of boredom or fear)	[27,28,29]
	UK	− (Increased consumption of energy-dense snacks)	[30]
	Italy	− (Increased consumption of unhealthy foods due to higher levels of anxiety)	[8]
	USA, China	− (Food as an element to reduce stress and cope with boredom)	[23]
	USA (Texas), China	− (Weight gain and reduced physical activity)	[31,32]
	USA, Poland, France, China, Brasil	− (Reduced physical activity and increased food intake, including snacks and ultra-processed foods)	[27,32,33,34,35]
Changes in consumer purchasing behavior and habits	UK, Spain, Great Britain (England, Scotland, Wales), China, Canada	− (Large-scale food stockpiling; panic buying)	[25,36,37,38,39]
	UK, Canada, USA	− (Intense short-term pressure on the whole supply chain, affecting the shortage of basic food products in stores)	[21,36,39]
	France	− (Purchasing fewer fresh products)	[27]
	USA	− (Increased consumer concern about the availability of groceries when shopping)	[7]
Changes in consumer behavior and habits	Canada, Kuwait	+ (Switching from food services to cooking at home)	[17,39]
	Turkey	+ (Increased consumption of foods that boost the immune system)	[40]
	USA, China	+ (Families spending more time cooking and eating together at home)	[23]
	USA, China, Kuwait	+ (Increased efficiency in the use of food when cooking at home and less waste)	[17,23]
	USA, China	+ (Closer family bonding around the dinner table and, perhaps, better nutrition from increased time spent on meal planning and preparing at home)	[23]
	Kuwait	+ (Taking actions by consumers to better manage food at home, for example, creating shopping lists and creative meal preparation)	[17]
Changes in food supply channels	UK, USA	+ (A large rise in non-store (online) sales)	[21,36]
	UK	+ (Rise of digital grocery shopping, which can result in a healthier overall shopping cart, as marketing and in-store promotions have less impact on consumers)	[41]
	USA, China	+ (Increased grocery shopping with curbside pickup or home delivery)	[23]

In the table, + shows a positive influence and − shows a negative influence.

**Table 2:** Impact of the COVID-19 pandemic on price changes, fluctuations in consumption and purchase of different meat types in selected countries.

Country/Region	Meat Type	Type of Impact	Fluctuations	Impact Timing	References
European countries	All meat categories	Price changes	↑	March–April 2020	[42]
			Prices stabilized	May 2020	
Poland	Beef Pork shoulder Bacon	Price changes	↑ 3.0% ↑ 20.6% ↑ 16.3%	First half of 2020 vs. first half of 2019	[43]
	Ribs		↑ 14.9%		
	Sausage		↑ 18.5%		
USA	Beef cuts (round, brisket, loin strips, chuck) Beef primal cuts (tenderloin, rib eye)	Price changes	↑ 39.1% ↓ 42%	6 March–10 April 2020	[44,45]
	Beef cuts (round, brisket, loin strips, chuck)		↑ 150%	10 April–8 May 2020	
			↑ 26.2%	May 2020–June 2020	
USA (South Carolina)	Ground beef	Price changes	↑ 100%	May 2020 from its mid-March price	[46]
	All meat categories		↑ 21.7%	The end of May 2020 (year-over-year)	
	Chicken		↑ 10.5%		
	Pork		↑ 17.7%		
China (Beijing, Shandong, and Hubei)	Pork	Price changes	↑	1 January–8 April 2020 vs. the same period in 2019	[47]
Turkey	Chicken	Price changes	↑	The end of 2020 and 2021	[48]
Turkey	Red meat Poultry	Consumption changes	↑ 10% ↑ 13%	May 2020	[49]
	Red meat		↓ 8%		
	Poultry		↓ 11%		
Poland	Canned meat Red meat	Consumption changes	↑ ↓	Before vs. during lockdown	[50]
China	Poultry (e.g., chicken, duck, goose) Red meat (e.g., beef, lamb, pork)	Consumption changes in urban areas	↑ 19.9% ↓ 5.6%	2020 vs. 2015–2019	[51]
	Poultry Red meat	Consumption changes in rural areas	↑ 34.6% ↓ 9.3%		
Slovakia	Meat Plant foods	Changes in sales	↓ ↑	During the pandemic	[52]
Slovenia	Fresh meat	Consumption changes	↓	Spring of 2020	[53]
Slovakia	Processed meat—ham, salami	Preference changes—women	↑ 22.40% ↓ 18.93%	During the pandemic	[54]
		Preference changes—men	↑ 28.10% ↓ 12.42%		
Italy, USA (New York State)	White meat Cold cuts Red meat Pork	Consumption changes	↑ ↑ ↓ ↓	During the lockdown compared to before the pandemic period	[55]
USA (South Carolina)	Local meat	Changes in local meat purchasing for home consumption	↑21.6% ↓ 20.4% decreased or stopped consumption	February 2020–November 2020	[46]
		Changes in local meat purchasing at restaurants	↑9.8% ↓ 38.5% decreased or stopped consumption		
Great Britain	Meat and fish	Changes in purchasing behavior	↑	During the lockdown	[56]
USA	Red meat—fatty cuts of meat or preparations such as hamburgers, steaks, lamb or pork chops	Consumption changes Women	↑ 2.9%	Pre-COVID-19 time period (5–11 March 2020) vs. during the first week of the COVID-19 lockdown (12–18 March 2020)	[57]
		Men	↑ 2.5%		
	Processed meat/sausage—fatty sausage, bacon, and other highly processed meats	Women Men	↓ 0.9% ↓ 1.8%		
	Lean protein—lean cuts of pork and beef, chicken, seafood, soy-based foods, protein shakes	Women Men	↓ 3.9% ↓ 3.6%		
European countries: Spain, Poland, Greece, Romania, UK, France, Germany, Finland, Sweden, Italy	Poultry	Consumption changes	↑ 21%	February 2020–September 2020	[58]
	Meat	All countries—expected France and Germany	↑ 19%		
		France	↓ 19%		
		Germany	↓ 17%		

In the table, ↑ shows an increase and ↓ shows a decrease.

## Data Availability

The raw data supporting the conclusions of this article will be made available by the authors on request.
